# CD47-targeted cancer immunogene therapy: Secreted SIRPα-Fc fusion protein eradicates tumors by macrophage and NK cell activation

**DOI:** 10.1016/j.omto.2021.09.005

**Published:** 2021-10-01

**Authors:** Magdalena Billerhart, Monika Schönhofer, Hemma Schueffl, Wolfram Polzer, Julia Pichler, Simon Decker, Alexander Taschauer, Julia Maier, Martina Anton, Sebastian Eckmann, Manuel Blaschek, Petra Heffeter, Haider Sami, Manfred Ogris

**Affiliations:** 1 University of Vienna, Faculty of Life Sciences, Department of Pharmaceutical Sciences, Laboratory of MacroMolecular Cancer Therapeutics (MMCT), Althanstrasse 14, 1090 Vienna, Austria; 2 Institute of Cancer Research, Department of Medicine I, Comprehensive Cancer Center, Medical University of Vienna, 1090 Vienna, Austria; 3 Institutes of Molecular Immunology and Experimental Oncology, Klinikum rechts der Isar, Technische Universität München, Ismaninger Straße 22, 81675 Munich, Germany

**Keywords:** gene therapy, polyplex, transfection, immunogene therapy, Fc receptor, macrophage, NK cells, CD47, signal regulatory protein alpha

## Abstract

CD47 protects healthy cells from macrophage attack by binding to signal regulatory protein α (SIRPα), while its upregulation in cancer prevents immune clearance. Systemic treatment with CD47 antibodies requires a weakened Fc-mediated effector function or lower CD47-binding affinity to prevent side effects. Our approach combines “the best of both worlds,” i.e., maximized CD47 binding and full Fc-mediated immune activity, by exploiting gene therapy for paracrine release. We developed a plasmid vector encoding for the secreted fusion protein sCV1-hIgG1, comprising highly efficient CD47-blocking moiety CV1 and Fc domain of human immunoglobulin G1 (IgG1) with maximized immune activation. sCV1-hIgG1 exhibited a potent bystander effect, blocking CD47 on all cells via fusion protein secreted from only a fraction of cells or when transferring transfection supernatant to untransfected cells. The CpG-free plasmid ensured sustained secretion of sCV1-hIgG1. In orthotopic human triple-negative breast cancer in CB17-severe combined immunodeficiency (SCID) mice, *ex vivo* transfection significantly delayed tumor growth and eradicated one-third of tumors. In intratumoral transfection experiments, CD47 blockage and increased migration of macrophages into the tumor were observed within 17 h of a single injection. Natural killer (NK) cell-mediated lysis of sCV1-hIgG1-expressing cells was demonstrated *in vitro*. Taken together, this approach also opens the opportunity to block, in principle, any immune checkpoints.

## Graphical abstract

**Figure:**
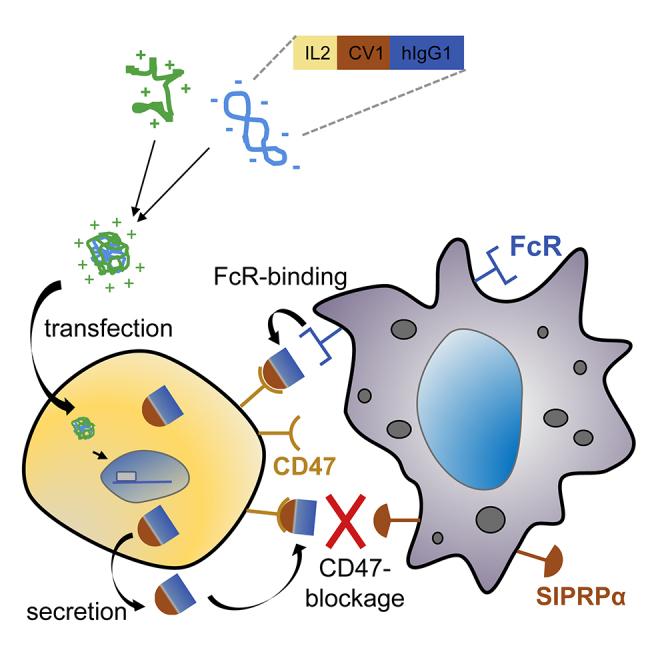

## Teaser

Here we present a novel gene therapy vector encoding for a secreted fusion protein, which blocks the “don’t eat me” signal CD47 in tumors and activates immune cells by its Fc domain. Highly efficient secretion, CD47 blocking, and tumor eradication by immune cell activation are demonstrated.

## Introduction

**Figure 1:**
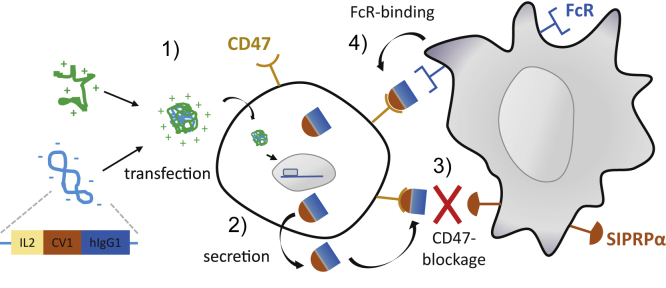
Schematic representation of CD47-targeted cancer immunogene therapy approach (1) Polyplexes formed by linear polyethylenimine (LPEI) and plasmid DNA encoding for sCV1-hIgG1 are internalized by tumor cells via adsorptive endocytosis; (2) sCV1-hIgG1 fusion protein is expressed and secreted into the intercellular space; (3) sCV1-hIgG1 blocks CD47 on transfected and adjacent tumor cells and inhibits its interaction with SIRPα on immune cells; (4) the Fc part of sCV1-hIgG1 binds to Fc receptors on macrophages, dendritic cells (DCs), neutrophils, and NK cells, activating both the innate and the adaptive immune system; this together allows the eradication of tumor cells by immune effector cells.

The immune system is a key component in growth control and eradication of cancer cells. Immune surveillance of tumors is divided into three phases, designated elimination, equilibrium, and escape.^1^ Tumor escape is promoted by the occurrence of an immune-suppressive microenvironment, which can also be triggered by hypoxia followed by localized expression of immune-suppressive cytokines like transforming growth factor-beta. Attraction of immune cells with suppressive functions, like regulatory T cells or M2 macrophages, then leads to the inhibition of natural killer (NK) and CD8^+^ cytotoxic T lymphocyte function.^2^ Body cells can overexpress surface proteins with immunomodulatory functions: Programmed Cell Death-Ligand 1 (PD-L1) expression in heart prevents T cell-mediated inflammation. However, PD-L1 overexpression on tumor cells and its interaction with programmed cell death 1 (PD-1) receptor on T cells suppresses their inflammatory and hence antitumoral response.^3^ CD47 (integrin-associated protein [IAP]), a ubiquitously expressed transmembrane glycoprotein, acts as a “marker of self” and plays an important role in hematopoiesis. Upon binding to CD47 ligand signal regulatory protein α (SIRPα), expressed on macrophages and dendritic cells, phagocytosis of target cells is inhibited.^4^ Also on erythrocytes CD47 prevents their SIRPα-mediated phagocytosis by macrophages, and loss of CD47 during senescence triggers their elimination. In hematopoietic cancers and also many solid tumors, CD47 is found to be upregulated and correlates with a poor prognosis.^5^ Also in cancer stem cells, the transmembrane protein level is increased.^6^ Therapeutic antibodies have proven to be an effective approach by specifically blocking a target on tumor cells and at the same time attracting and activating immune cells by the exposed Fc part triggering antibody-dependent cell-mediated cytotoxicity (ADCC). Cetuximab, for example, blocks the epidermal growth factor receptor (EGFR) and subsequent downstream signaling, whereas its immunoglobulin G (IgG)1-derived Fc successfully triggers both ADCC and complement activation (complement-dependent cytotoxicity [CDC]).^7^ As EGFR is often overexpressed on cancer cells (compared to normal expression levels on non-malignant tissue), this allows a certain selectivity for the antibody treatment. In contrast, CD47 expression is abundant in all tissues and particularly high in erythrocytes. This poses the risk of an “antigen sink” by losing most of the CD47 antibody, and it can trigger severe anemia as a side effect.^5^ Several approaches are on the way to find a balance between the binding affinity for CD47 and triggering ADCC and/or CDC. High-affinity CD47-binding antibodies like Hu5F9-G4 (magrolimab) and SRF231 are being evaluated in clinical trials or are already approved, and both are based on IgG4. They show reduced complement activation and ADCC but also fewer side effects.^5^^,^^8^ As an alternative, fusion proteins have been developed that block CD47 with lower affinity but induce more potent immune activation. TTI-621 is a fusion protein of SIRPα and the Fc domain of human IgG1.^9^ Although the binding constant for CD47 with TTI-621 is significantly lower than that of anti-CD47 antibodies (5F9: k_D_ = 8 nM;^10^ human SIRPα: 300–1.000 nM^11^), the IgG1 Fc shows potent macrophage activation and ADCC but avoids erythrocyte agglutination.^12^ Recently, Trillium Therapeutics also developed an alternative, TTI-622, with similar a SIRPα domain but an IgG4 Fc domain, in an attempt to improve tolerability of the protein (ClinicalTrials.gov Identifier: NCT03530683). Weiskopf and colleagues designed a high-affinity variant of SIRPα, termed CV1, with a binding constant of 11 pM for human CD47.^13^ When a CV1-hIgG4 Fc fusion protein was applied, a significantly decreased hematocrit in NSG mice was observed. Full antitumoral activity in the absence of toxicity was achieved by application of the CV1 monomer combined with, e.g., rituximab in CD20-positive lymphoma xenografts. The fusion protein ALX148 was developed, which combines a high-affinity SIRPα version with an IgG Fc part lacking any CDC or ADCC activity.^14^ In syngeneic and xenograft tumor models, the combination of ALX148 with PD-L1 or CD20-blocking antibodies was necessary for potent antitumoral activity. In non-human primates, ALX148 alone was non-toxic up to the highest dose tested, i.e., 100 mg/kg. Both TTI-621 and ALX148 are already evaluated in clinical trials to treat a rare T cell lymphoma^15^ or advanced head and neck cancer in combination with pembrolizumab (Kim et al., 2019, Am. Soc. Hematol., abstract). We and others have demonstrated that the tumor-restricted expression of secreted therapeutic proteins can be a vital approach, which also allows a potent bystander effect on adjacent, non-transfected cells in the near vicinity. With a synthetic gene delivery system, systemic application of plasmid vector resulted in significant tumor necrosis factor alpha (TNF-α) expression in implanted tumors.^16^ Interleukin-12 (IL-12), a highly potent cytokine with antitumoral activity, has been expressed in tumors after local delivery and is being investigated already in several clinical trials.^17^ For our present study, we designed a gene vector encoding for a secreted fusion protein having high-affinity SIRPα variant CV1 coupled to a IgG1 Fc part with maximized CDC and ADCC activity. Secretion is accomplished by the IL-2 secretion signal, which resulted in a potent bystander effect even at very low transfection efficiencies. This enables selective blocking of CD47 in cancer cells and finally leads to immune cell activation and tumor cell eradication (Figure 1).

## Results

### Design and cloning of a gene vector for secreted sCV1-hIgG1 fusion protein

We have designed a set of plasmids, namely therapeutic plasmid psCV1-hIgG1 with dual/combined functionality of CD47 blockage and Fc-mediated cytotoxicity, single-component control plasmids, and transfection control plasmid (for plasmid maps see Figure S1). All of these plasmids are based on the CpG-free backbone from pCpG-hCMV/SCEP-mCherry, to ensure optimal transgene expression *in vitro* and *in vivo*.^18^ As further controls to the therapeutic plasmid (psCV1-hIgG1), plasmids were cloned that encode for either secreted hIgG1-Fc only (pshIgG1) or secreted SIRPα CV1 variant only (psCV1), or as a transfection control the fluorescent reporter mCherry (pCpG-hCMV/SCEP-mCherry, short: pmCherry) was used. All plasmids could be propagated as low-copy plasmids in *E. coli* DB3.1 λpir or GT115. With the designed plasmid at hand, we could produce ∼3 mg of plasmid per liter of culture volume.

### sCV1-hIgG1 exhibits combined CD47 blockage and Fc functionality and potent bystander effect *in vitro*

**Figure 2:**
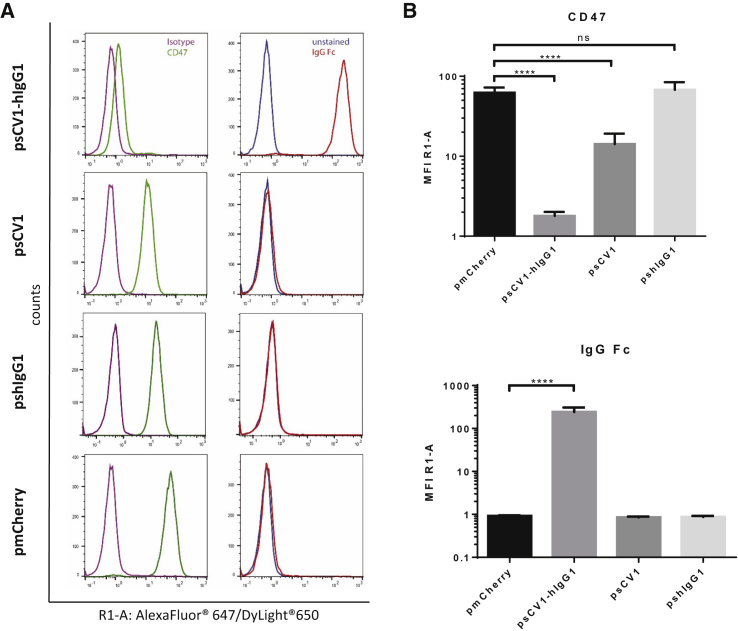
CD47-blockage and sCV1-hIgG1 binding after *in vitro* transfection 231/LM2-4 cells were transfected in T-75 flasks with 40 μg of indicated plasmid for 4 h in basal medium (thereafter same amount of complete medium added), harvested after 48 h, and analyzed by flow cytometry. CD47 blockage was evaluated by staining with anti-CD47 antibody clone B6H12.2 competing with sCV1-hIgG1 for CD47 binding and isotype IgG staining as control (secondary antibody goat anti-mouse IgG H&L Alexa Fluor 647). sCV1-hIgG1-binding to the cell surface was detected by goat anti-human IgG Fc-DyLight 650 staining or goat IgG isotype-AF647 conjugate. (A) Histogram plots. Left: CD47 stain. Right: IgG-Fc stain. (B) Average signal intensity (geometric mean of individual measurements). Top: CD47 staining. Bottom: IgG-Fc staining. n = 12 + standard deviation (SD). ∗∗∗∗p ≤ 0.0001, ns p > 0.05; U test (Mann-Whitney).

**Figure 3:**
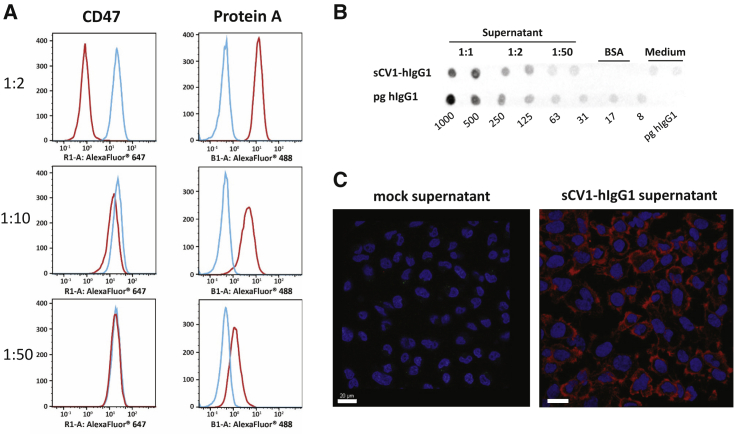
Bystander effect and fusion protein secretion *in vitro* 231/LM2-4 cells were transfected for 48 h, and the supernatant was harvested thereafter and either analyzed for fusion protein content or transferred onto untransfected 231/LM2-4 cells. (A) Cells treated with transfection supernatant for 1 h (diluted with complete medium at the indicated ratio) and thereafter stained with anti-CD47 antibody [B6H12.2] (secondary antibody goat anti-mouse IgG H&L Alexa Fluor 647, left) or protein A (Alexa Fluor 488 conjugated, right). Representative histogram blots shown. Red line, cells incubated with supernatant from psCV1-hIgG1-transfected cells; blue line, cells incubated with supernatant from mock-transfected (buffer only) cells. (B) Dot blot analysis (2 μL/dot) with anti-human IgG Fc HRP antibody. Top (dots in duplicates): 48-h transfection supernatant diluted with complete medium at the indicated ratio, BSA solution (2 mg/mL), complete medium. Bottom: indicated amounts per dot of human IgG1 isotype. (C) Cells treated with 48-h transfection supernatant for 1 h (right) or supernatant from mock-transfected (buffer only) cells (left), washed, fixed, and stained with DAPI (blue) and goat anti-human IgG Fc (DyLight 650). Confocal laser scanning microscopy (CLSM) imaging was conducted with a 40× oil objective; representative middle sections of the cells are shown. Scale bars: 20 μm.

**Figure 4:**
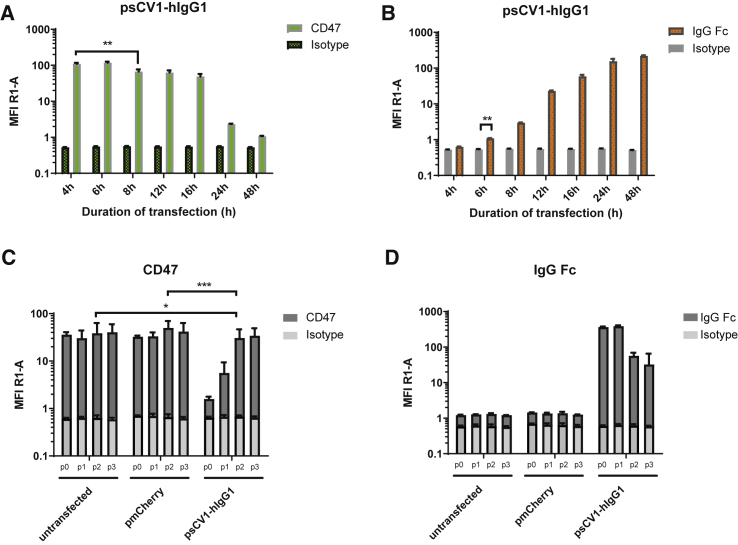
Time course of transgene expression *in vitro* 231/LM2-4-EGFPLuc cells were transfected (A and B) in T-75 flasks with psCV1-hIgG1 or (C and D) in T-25 flasks with psCV1-hIgG1 or pmCherry or mock transfection (buffer only) for 4 h in basal medium, and thereafter the same amount of complete medium was added. Cells were harvested and analyzed at (A and B) the indicated time point or (C and D) 48 h after transfection (p0) and passaged further (ratio 1:8) on day 5 (p1), day 9 (p2), and day 12 (p3) into new T-25 flasks with fresh complete medium, and remaining cells were analyzed by flow cytometry. (A and C) Cells stained with anti-CD47 antibody [B6H12.2] or IgG isotype (secondary antibody goat anti-mouse IgG H&L Alexa Fluor 647). (B and D) Cells stained with goat anti-human IgG Fc-DyLight 650 or goat IgG isotype AF647 conjugate. n = 6 +SD. ∗p < 0.05; ∗∗p < 0.01, ∗∗∗p < 0.001; U test (Mann-Whitney).

Before investigation of psCV1-hIgG1 transfection and fusion protein functionality, expression of CD47 was measured by flow cytometry on human lung and triple-negative breast carcinoma cell lines A549 and MDA-MB-231 and their metastatic variants 231/LM2-4 and 231/LM2-4-EGFPLuc, respectively (Figure S2). All of them were clearly positive for CD47, with MDA-MB-231 cells exhibiting a higher level compared to A549. The screened cell lines were then transfected with plasmid psCV1-hIgG1, encoding for the secreted fusion protein combining CD47 blockage and Fc functionality, and other control plasmids for deciphering the presence of combined/dual functionality. We measured both blocking of CD47 (with a competitive anti-CD47 binding assay) and the presence of human IgG1-Fc domain (with an anti-IgG1 antibody on the cell surface) as shown in Figure 2. With both readouts we observed an effect on all measured cells, i.e., a complete shift of the whole cell population. When stained for the Fc part of the fusion protein, a strong shift in signal was observed on psCV1-hIgG1-transfected cells, whereas no signal above background was observed for psCV1- and pmCherry-transfected cells. Blockage of CD47 binding was most pronounced on psCV1-hIgG1-transfected cells, with an ∼96% signal reduction. Of interest, on psCV1-transfected 231/LM2-4 cells a partial block of 76% was observed, whereas on A549 cells CD47 access for the antibody was blocked to a similar extent on psCV1-hIgG1- and psCV1-transfected cells (Figure S3). Using mCherry as a reporter plasmid, we observed under similar transfection conditions only 1%–2% mCherry-positive cells 48 h after transfection of 231/LM2-4-EGFPluc cells when analyzed by flow cytometry (Figure S4), whereas A549 cells could be transfected with an efficiency of >28% (data not shown). In view of the apparently strong bystander effect and to further substantiate the specificity, we next evaluated CD47 blockage and fusion protein detection as a function of the amount of transgene expressed and the effect of diluted supernatant (from psCV1-hIgG1-transfected cells) transferred to non-transfected recipient cells (Figure 3). CD47 blockage was evaluated similarly as in Figure 2, whereas cell surface-bound sCV1-hIgG1 protein was measured by flow cytometry with fluorescently labeled protein A (which binds to the Fc domain). With a 1:2 diluted supernatant from transfected cells, CD47 access was strongly blocked in all recipient cells, whereas with 1:10 diluted supernatant a minimal effect was seen, and none was seen with the 1:50 dilution (Figure 3A). Similar as for the anti-IgG Fc staining, protein A also staining allowed sensitive detection of Fc-positive cells even when treated with a 1:50 diluted supernatant. From a dot blot assay (Figure 3B) and a respective calibration curve, we estimated a concentration of sCV1-hIgG1 in the supernatant corresponding to ∼0.24 ng/μL IgG protein, which corresponds to a concentration of 3.1 nM, produced within 48 h after initial transfection. The amount secreted was also sufficient to be detected by immunostaining of transfection supernatant-treated, untransfected cells (Figure 3C). Since the CV1 sequence was initially optimized for binding to human CD47, we also transfected a murine cell line to evaluate the protein for its binding affinity for murine CD47 (Figure S5), as Weiskopf et al. also demonstrated cross-reactivity with mouse CD47.^13^ Similar as for the two human cell lines evaluated, we could also detect efficient surface binding of fusion protein in the murine colon carcinoma cell line CT26 when staining the Fc part with fluorescently labeled protein A. Next, we studied the time course of *in vitro* transgene expression for sCV1-hIgG1 within two different experimental setups, one over a time frame of 4–48 h and the other from 2 to 12 days. Earliest detectable CD47-blocking activity could be observed 6 h after transfection, although after 48 h it was most pronounced (Figure 4A; Figure S6). Surface-bound sCV1-hIgG1 could already be detected at 6 h after transfection, and the signal increased up to 48 h (Figure 4B; Figure S6). When psCV1-hIgG1-transfected cells were tracked over days, CD47 blocking was still measurable after two 1:8 passaging steps (Figure 4C, p1 and p2). With an antibody directed against IgG-Fc, detection of bound sCV1-hIgG1 protein was even possible also at passage p3, i.e., after 12 days of transfection (Figure 4D). This is noteworthy considering an initial transfection rate below 1.5% (see Figure S4) and with far less than 1% of cells expressing sCV1-hIgG1 protein over the investigated duration (Figures 4C and 4D).

### Therapeutic activity *in vivo*

**Figure 5:**
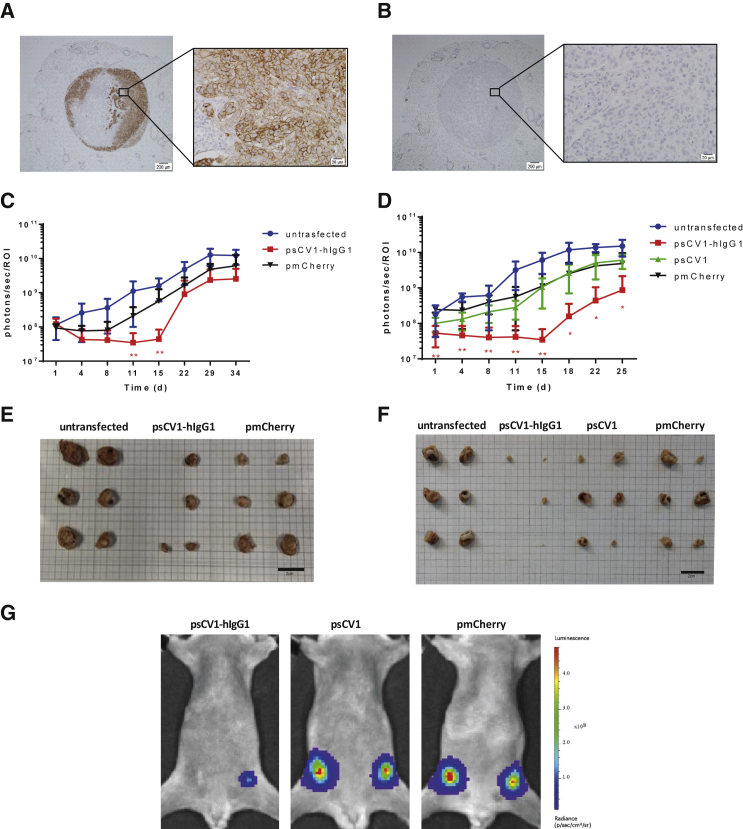
psCV1-hIgG1 *ex vivo* transfected tumor cells show significantly reduced growth *in vivo* (A and B) CD47 expression in a 231/LM2-4-EGFPLuc lymph node metastasis 34 days after orthotopic implantation with (A) huCD47 staining and (B) isotype control staining; 4× magnification images with zoomed-in 40× magnification images. Scale bars: 200 μm (4× magnification) and 20 μm (40× magnification. (C–G) 231/LM2-4-EGFPLuc were transfected *ex vivo* with the indicated plasmid and 48 h thereafter orthotopically implanted into the 4^th^ pair of inguinal nipples (2 sites/animal) in female CB17-SCID mice. Two experiments [Exp I (C and E) and Exp II (D, F, G)] were conducted. Exp I was terminated 34 days after implantation and Exp II after 25 days. 3 animals/group were used, and the BLI signal at each injection site was quantified within a separate ROI (i.e., n = 6 +SD per group). (C and D) Tumor growth followed by BLI. (E and F) Size of explanted tumors after termination. (G) Representative BLI pictures (color coded, overlaid onto a reflected light picture) of animals in Exp II 18 days after implantation. (C and D): n = 6 per experiment, ∗∗p ≤ 0.01, ∗p ≤ 0.05; psCV1-hIgG1 transfected versus pmCherry transfected; U test (Mann-Whitney). Scale bars in E and F: 2 cm.

**Figure 6:**
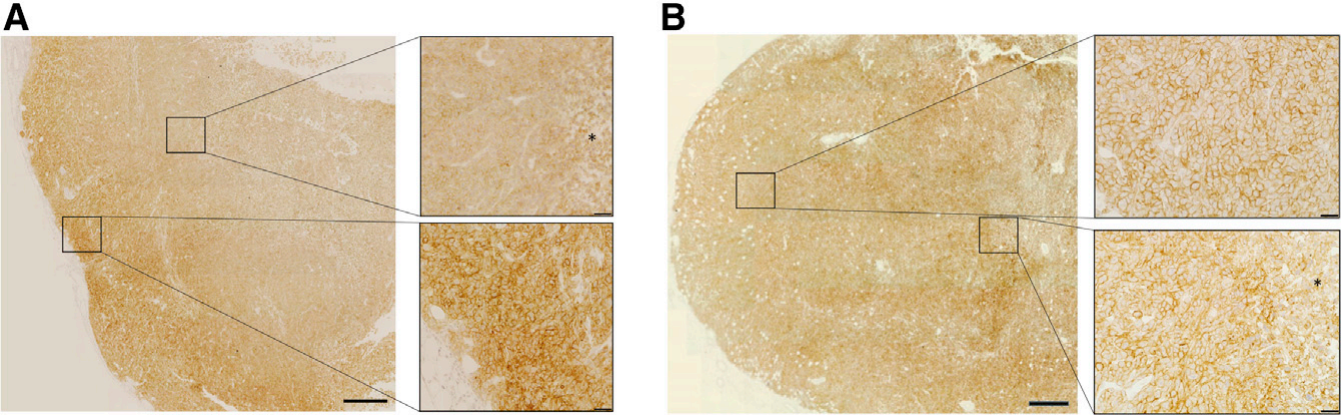
CD47-blockage in polyplex-transfected tumors 13-day-old, palpable 231/LM2-4-EGFPluc tumors were transfected with LPEI-based nanoparticles loaded with psCV1-hIgG1 or pmCherry at N/P ratio 9 by injection into the middle of the tumor core (from two puncture sides per tumor). Tumors were harvested 24 h after injection and stained for huCD47. (A) Gradient CD47 staining increasing from the injection site to the periphery of a psCV1-hIgG1-transfected tumor. (B) CD47 staining of a pmCherry-transfected tumor. Asterisk, necrotic area. Image stitching with zoomed-in images at 40×. Scale bars: 2 mm (stitched overview pictures), 20 μm (zoomed-in pictures).

**Figure 7:**
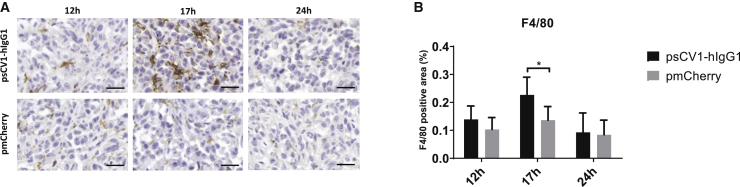
Intratumoral psCV1-hIgG1 transfection triggers macrophage infiltration Macrophage staining of *in vivo* transfected 231/LM2-4-EGFPLuc tumors. (A) IHC staining with an anti-F4/80 antibody murine macrophage marker of tumor tissue harvested 12 h, 17 h, or 24 h after *in vivo* transfection by intratumoral injection of polyplexes formulated with psCV1-hIgG1- or pmCherry-expressing pDNA into 13-day-old tumors; 40× magnification, scale bar: 25 μm. (B) Semiquantitative analysis of macrophage staining signal shown in bar charts (in %); n = 4–6 + SD. ∗p < 0.05; U test (Mann-Whitney).

Next we evaluated the effect of sCV1-hIgG1 gene therapy in an orthotopic xenograft model of MDA-MB-231 human triple-negative breast cancer in CB17-severe combined immunodeficiency (SCID) mice (Figure 5). When implanting the highly malignant metastatic variant 231/LM2-4-EGFPLuc percutaneously into the 4^th^ pair of inguinal nipples, we observed, besides fast onset of local growth, axillary and sentinel lymph node infiltration being positive for CD47 (Figures 5A and 5B). In two independent experiments with the same settings, 231/LM2-4-EGFPLuc were transiently transfected *ex vivo* 48 h prior to implantation with pmCherry, psCV1, or psCV1-hIgG1 or left untransfected and thereafter implanted. Tumor growth was semiquantitatively followed by bioluminescence imaging (BLI), and tumors were explanted at the end of the experiment. Whereas in experiment I measurements were continued until day 34, when animals in the untransfected tumor group reached endpoint criteria (reduced well-being, which was also due to metastasis), in experiment II the study was terminated and tumors explanted on day 25 after implantation. In the case of untransfected cells, the BLI signal doubled approximately every 4 days starting with implantation. In both experiments, psCV1-hIgG1-transfected tumors showed a significant, on average 50% reduced signal for >2 weeks after implantation, compared to the untransfected control group (Figures 5C and 5D). In individual tumors, this reduction was down to 0.1% of the starting signal. After day 15, re-growth started in some tumors, whereas at the end of the experiment (day 25 or 34 after implantation, respectively), only four of six (8 of 12 in total) implanted tumors could be detected in both experiments, with sizes ranging from 1 to 4 mm in diameter (Figures 5E and 5F). Although marked with luciferase, no sCV1-hIgG1-transfected tumors could be detected by BLI (representative BLI picture in Figure 5G) in two out of six mice (4 out of 12 in total). The growth curve of the psCV1 tumors was rising similar to that of the pmCherry transfection control group (Figure 5D). Also, the tumor size after harvesting was correlating (Figure 5F). However, the growth curve of the untransfected cells showed a slightly higher growth tendency compared to pmCherry and psCV1 tumors, which could be due to the intrinsic growth retardation effect of the *ex vivo* transfection process in the latter case. Nevertheless, after termination of the experiment pmCherry-transfected tumors (6/6 per experiment, 12/12 in total) were all large (diameter 10–15 mm) and showed signs of core necrosis, whereas untransfected tumors were exceeding a diameter of 20 mm (Figures 5E and 5F). We also quantified the volume of explanted tumors (Figure S7). In Exp I (terminated on day 34 after implantation), pmCherry-transfected tumors were significantly smaller than the untreated control and psCV1-hIgG1-transfected tumors were significantly smaller than the pmCherry-transfected tumors (Figure S7A). When terminating the experiment on day 25 (Exp II), the size of pmCherry-transfected tumors was slightly, but insignificantly, smaller compared to untransfected tumors (Figure S7B). Nevertheless, only sCV1-hIgG1-transfected tumors were significantly smaller then pmCherry-transfected tumors. The implantation of *ex vivo* transfected tumor cells could demonstrate that tumor-localized expression of sCV1-hIgG1 had a therapeutic effect. Furthermore, we were also interested in whether the plasmid vector is in principle suitable for a local therapeutic approach by inducing localized immune reaction and CD47 blockage. When forming plasmid-linear polyethylenimine (LPEI) polyplex particles with a positive charge excess (N/P ratio 9) in a low-ionic buffer (5% glucose, 20 mM HEPES pH 7.4 [HBG]), we usually obtained particles sized ∼120 nm in diameter (mode) and ζ-potential of approximately +30 mV,^19^ which were then injected intratumorally in 231/LM2-4-EGFPluc tumors. In a first series, tumors were explanted 24, 48, and 72 h after *in vivo* transfection. When comparing the different time points, at 24 h after transfection we observed areas of blocked CD47 in immunohistochemical (IHC)-stained slides, whereas such blockage was not seen in the control transfected tumors (Figure 6). Of interest, no blockage was observed at 48 and 72 h after transfection with psCV1-hIgG1. In a further *in vivo* series tumors were explanted 12, 17, and 24 h after intratumoral polyplex injection, and macrophage infiltration was qualitatively and semiquantitatively analyzed by staining for the murine macrophage antigen F4/80 (Figure 7). Although the level of macrophage infiltration was higher at all time points in psCV1-hIgG1-transfected tumors compared to the pmCherry-transfected control group, only at 17 h after transfection did psCV1-hIgG1-transfected tumors show significantly elevated macrophage infiltration. This early time point is also in line with the *in vitro* findings, where secretion of sCV1-hIgG1 was detected already after 6 h of transfection (Figure 4).

### NK cell activation

**Figure 8:**
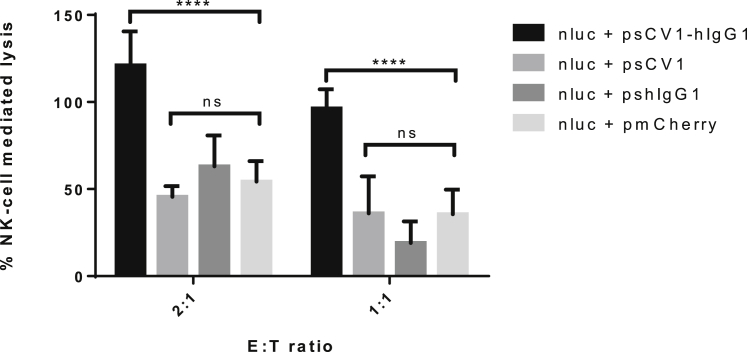
sCV1-hIgG1 enhances NK cell-mediated tumor cell lysis 231/LM2-4 cells were co-transfected with plasmid pNluc (40 μg) and the indicated plasmid (psCV1-hIgG1, psCV1, pshIgG1, pmCherry; 40 μg) in T-75 flasks for 48 h, transferred to 48-well plates, and 24 h thereafter co-cultured with NK92 cells at the indicated E:T ratio for 4 h. Cells treated with medium containing 0.1% Triton X-100 served as positive control (100%) and cells without co-culture as negative control (0%). Nluc activity was quantified in the supernatant. n = 9 + SD. ∗∗∗∗p < 0.0001, ns p > 0.05; U test (Mann-Whitney).

To detect potential infiltration of tumors with NK cells, sections were stained with anti-natural cytotoxicity triggering receptor 1 (NCR1) antibody, which is exclusively expressed on NK cells. Unfortunately, we could neither verify nor falsify this hypothesis in our model, as all NK cell antibodies tested in our laboratory resulted in high background staining, which did not allow detection of any differences. Therefore, to study the effect of sCV1-hIgG1 on human NK cells *in vitro*, we developed a luciferase-based *in vitro* assay that should allow monitoring of NK cell-mediated target cell lysis *in vitro*. NK cell-mediated lysis of luciferase-expressing tumor cells co-transfected with desired plasmid allowed investigation of the role of NK cells in CD47 blockade and opsonization via sCV1-hIgG1. In a preceding experiment, the lysis activity of the NK cell line against pNLuc-transfected 231/LM2-4 cells was evaluated at increasing E:T ratios (Figure S8). NK cell-mediated lysis at E:T ratios of 1:1 and 2:1 was studied to investigate the role of NK cells in CD47 blockade and opsonization with sCV1-hIgG1 (Figure 8). Although psCV1 or pshIgG1 transfection did not induce any cell lysis above the level of transfection control (pmCherry), psCV1-hIgG1-transfected 231/LM2-4 cells were efficiently lysed at E:T ratios of 2:1 and 1:1.

## Discussion

Our approach here was to combine “the best of both worlds,” i.e., maximized binding affinity for human CD47 combined with full CDC and ADCC activity. Although this would be almost impossible when applying systemic protein therapy, we developed a gene therapy approach for tumor-directed transgene expression and secretion of the therapeutic protein directly by the tumor cells. We evaluated sCV1-hIgG1 expression, secretion, and binding to CD47, as well as its bystander effect *in vitro*, and confirmed the efficacy of this therapeutic plasmid *in vivo*.

In our plasmid design, we utilized the CpG-free shuffled CMV-EF1 promoter (SCEP), which combines elements of the strong constitutive cytomegalovirus (CMV) promoter and the human elongation factor-1-alpha promoter and, when used in combination with the hCMV enhancer element within a CpG-free plasmid backbone, enables high and sustained transgene expression levels.^20^ The high-affinity CV1 consensus sequence based on human SIRPα^13^ was fused with an Fc variant based on human IgG1 carrying a point mutation (E333A), which boosts both ADCC and CDC and exhibits increased binding affinity to Fc gamma receptor (FcγR)IIIa.^21^ Secretion was ensured with a 20-amino acid secretion signal sequence derived from human IL-2 with intracellular cleavage of the signal after serine at position 20. As a transfection reagent we utilized LPEI with a molecular weight (MW) of 10 kDa. This polymer allows the formation of nanosized, positively charged polyplexes, which enable cellular uptake by adsorptive endocytosis and promote endosomal release of the payload by a proton sponge mechanism.^19^ Both SCEP promoter and LPEI polyplex result in non-selective transfection and transgene expression. Here, tumor-specific delivery and tumor cell-specific transgene expression can be achieved, for example, by de-targeting approaches using microRNA (miRNA)-binding sites, which restrict transgene expression to cells lacking otherwise ubiquitously expressed miRNAs, e.g., miRNA 143.^18^ Similarly, the polyplex approach could be improved by shielding the positive charge with polyethylene glycol and attaching a cell-binding ligand, e.g., an EGFR-binding peptide, which promotes selective uptake of polyplexes in EGFR-overexpressing tumor cells.^16^ Prior to transfection studies, all cell lines were first screened for CD47 expression (Figure S2). Interestingly, the MDA-MB-231-derived subline 231/LM2-4, which was initially isolated from a lymph node metastasis after orthotopic tumor implantation into SCID mice,^22^ did not show any further elevated CD47 expression levels compared to the cell line it was derived from. For both A549 and MDA-MB-231, elevated CD47 levels have been reported and are considered to be a driver for metastasis for the respective tumor type (non-small cell lung cancer [NSCLC], A549 cells; triple-negative breast cancer, MDA-MB-231 cells).^23^^,^^24^ After transfection, CD47 blockage was determined with the anti-CD47 antibody clone B6H12.2, which was shown to have steric clashes with SIRPα and blocks its interaction with CD47,^25^ and the presence of sCV1-hIgG1 was detected with an anti-human IgG Fc antibody (Figure 2) or fluorescently labeled protein A (Figure 3). The fact that the whole cell population was positive for human IgG Fc, albeit only a fraction is expressing the transgene (as shown by mCherry as reporter), points to the efficient secretion of the protein due to the IL-2 secretion sequence^26^ and a potent bystander effect, when treating untransfected cells with transfection supernatant (Figure 3C). We also estimated the total amount of fusion protein secreted over time (Figure 3B). The fusion protein has a MW of 38.5 kDa (calculated at https://web.expasy.org/compute_pi/), with the monomeric CD47-binding SIRPα variant CV1 (13 kDa)^13^ fused to the IgG1 Fc region comprising the CH2 and CH3 domains of the IgG heavy chain and the hinge region (25.5 kDa). Because of the cysteine residues in the hinge region, the protein dimerizes and exhibits a final MW of 77 kDa. In relation to the concentration of IgG1 (∼150 kDa), this would correspond to a concentration of 0.12 μg/mL. It should be noted that this amount of secreted sCV1-hIgG1 is in addition to the amount already bound to CD47 on cells expressing the fusion protein; thus the total amount can be expected to be considerably higher. Nevertheless, compared to protein therapy, this amount appears rather low. In a preclinical setup, NSG mice xenografted with CD47-positive tumors were treated intravenously with a daily dose of 7.5 mg/kg CV1-hIgG4.^13^ This corresponds to an average blood concentration (assuming 8% blood content) of ∼90 μg/mL (i.e., >1 mM). With the therapeutic anti-CD47 antibody Hu5F9-G4, plasma levels of ∼1 mg/mL were achieved with dosages ranging from 20 to 45 mg/kg.^5^ Antibody accumulation in solid tumors is typically rather low, ranging from 0.001% to 0.01% of the injected dose per gram of tumor. Thurber and Weissleder developed a theoretical model to estimate absolute antibody concentration. Even at saturating concentrations, where >5,000 mg would be needed for a 70-kg patient to target a highly expressed antigen, intratumoral antigen concentration could be as low as 100 nM.^27^ Even then, the most rate-limiting step is extravasation and diffusion within the tissue. Hence, we assume that a relatively low dose of fusion protein continuously expressed in the tumor could in principle be sufficient for a potent antitumoral effect while at the same time preventing side effects in other organs. Although protein therapy relies on repeated dosing, local expression can be a continuous source of therapeutic protein. Evaluating both early and late time points after transfection for CD47 blocking activity and Fc detection (Figure 4; Figure S6), we observed both a fast onset (detectability of the Fc part 6 h after transfection) and prolonged duration (12 days and three passages after transfection; Figure 4) of expression. Therefore, one could at least estimate that a single plasmid transfection could last for >2 weeks, as experiments with electroporation of human tumors also revealed that transgene expression is found for >3 weeks.^17^

For *in vivo* evaluation, we have selected a xenograft model in CB17-SCID mice. With the EGFP-Luc labeled, orthotopically implanted 231/LM2-4-EGFPLuc cells we observed a fast onset of tumor growth (Figure 5), which is in line with observations by Munoz et al., where tumors of size >500 mm^3^ developed within 3 weeks and showed metastases in various areas, including lymph nodes and mesenterium.^22^ Similarly, we could demonstrate axillary lymph node infiltration being positive for CD47 (Figure 5A). In contrast, the parental cell line MDA-MB-231, also when labeled with EGFPLuc and implanted orthotopically, shows a very slow onset of tumor growth and can be followed via BLI for several months (M. Ogris et al., unpublished observations). In both experiments, psCV1-hIgG1-transfected tumors exhibited a rapidly decreasing BLI signal after implantation. Considering the BLI sensitivity, loss of BLI signal and absence of 4 out of 12 tumors after explantation also points to a complete tumor eradication, as with a similar BLI imaging setup even single-digit numbers of luciferase-expressing cells can be detected.^28^ Here, caliper-based size measurements would not be applicable to measure the presence of tumor cells. Also, BLI only detects metabolically active tumor tissue without considering a necrotic core. Still, pmCherry-transfected tumors appeared smaller then untreated tumors, which was significant in Exp I when measuring the size of explanted tumors (Figure S7). It has been reported that polycations like LPEI or cationic dendrimers can exert an intrinsic effect on delayed tumor cell growth, as observed by us (M. Ogris et al., unpublished observations) and others.^29^ In Exp II, psCV1-transfected tumors gave BLI signals similar to pmCherry-transfected tumors and were only insignificantly smaller. Apparently the CD47 block alone is insufficient for tumor growth retardation, which is in accordance with the findings of Weisskopf et al.: while applying the CD47-blocking protein CV1 monomer alone, no reduction of tumor growth *in vivo* was observed. Only with co-application of rituximab (in an EGFR-positive xenograft model), tumor growth was reduced and led to complete tumor eradication and long-term survival (>250 days) in 25% of treated animals.^13^ Thus, we conclude that also with a locally produced CD47-blocking protein, a functional Fc part for potential macrophage and/or NK cell activation is quite crucial and needed.

In principle, a syngeneic tumor model would also be applicable, as we also demonstrated sCV1-hIgG1 binding to murine CD47 on CT26 cells (Figure S5). This is in line with previous results, where ALX148, a fusion protein based on a high-affinity SIRPα version, acts antitumorally in combination with anti PD-L1 on implanted CT26 tumors in mice.^14^ Although murine SIRPα (from C57BL/6 mice) binds with similar affinity (k_D_ ∼5 μM) to human and murine CD47, a mutation in the SIRPα sequence in non-obese diabetic (NOD)/SCID mice exhibits an exceptional affinity for human CD47 with a k_D_ of 0.08 μM.^30^ Interestingly, human SIRPα binds human CD47 with a k_D_ of 0.3–0.6 μM, i.e., with a >8-fold higher affinity compared to mice. This points to a potentially higher impact of the SIRPα-CD47 axis in human physiology and oncology compared to mice and makes it necessary to use high-affinity CD47 binders to unleash the full therapeutic potential. Although *in vitro* transfected cells were used for proof of principle, local or systemic application of gene vectors is inevitable in a therapeutic setting. Also, intratumoral application of plasmid DNA is a vital option for the treatment of unresectable tumors or as a neoadjuvant treatment. For example, image-guided injections of LPEI polyplexes were used for the treatment of pancreatic ductal adenocarcinoma,^31^ where, in the case of larger tumors, multiple injections can also be necessary and are clinically feasible.^17^^,^^31^ Twenty-four hours after intratumoral transfection, we observed local blockage of CD47 surrounding the injection site in the millimeter range as shown by immunohistochemistry (Figure 6). This localized CD47 blockage can be explained by several means. First, we expect sCV1-hIgG1 to be quickly absorbed by transfected and adjacent tumor cells, as they express high amounts of CD47 (Figure 5A), limiting a further spread. Second, this can also be due to limited total amount of sCV1-hIgG1 fusion proteins produced by tumor cells after transfection, as our *in vitro* measurement on transfected 231/LM2-4 cells gave a value in the single nanomolar range (Figure 3B). This is 10^6^-fold lower compared to protein therapies, where 7.5 mg/kg of CV1-hIgG4 protein (corresponding to ∼1 mM) was applied.^13^ As we did not observe any toxicities in the treated animals, we expect these effects (local absorption after transfection, low concentration) also to be responsible for the absence of toxic side effects, which otherwise occurred in the case of systemic administration of a CV1-hIgG4 fusion protein (i.e., reduced hematocrit).^13^

Despite the low amount expressed, the antitumoral activity of sCV1-hIgG1 was significant (Figure 5), which could also be due to the hIgG1-Fc domain used. While the hIgG4 Fc part is known to have low ADCC and CDC activation potential, hIgG1 Fc has a 10-fold higher affinity for both human and murine FcγRI and a 2-fold higher affinity for FcγRIII (CD16), which is expressed on NK cells, neutrophils, and macrophages.^32^ We have utilized the hIgG1 version with a point mutation (E333A), which further increases complement activation^21^ and binding to FcγRIII.^33^ This, together with the fact that the fusion protein is produced locally without the need to diffuse toward tumor cells, can explain its similar efficiency *in vivo* compared to protein therapy, where dosages several orders of magnitude higher had to be used. We also aimed to unravel the role of macrophages after sCV1-hIgG1-mediated CD47 blocking *in vivo* and observed significantly increased tumor infiltration at 17 h after transfection (Figure 7). This would be in line with the literature, as recruitment of macrophages and their infiltration into tumor tissue appears rather fast after CD47 blockade: with a CD47-blocking antibody applied in an osteosarcoma xenograft model, significant macrophage infiltration was measurable 24 h after treatment.^34^ We did not distinguish between different subsets of macrophages, as we only stained for the pan-macrophage marker F4/80. Lin and colleagues demonstrated that CD47 blockade *ex vivo* with TTI-621 (a fusion protein of an engineered SIRPα protein and human IgG1-Fc) on lymphoma cells triggered their phagocytosis by a broad subset of macrophages, including both the M1 and the M2 subtype.^35^ Nevertheless, Mohanty and colleagues observed mainly M1 macrophage (F4/80^+^/CD80^+^) and not M2 macrophage (F4/80^+^/CD260^+^) infiltration after CD47 blockade *in vivo*.^34^ Still, as the quantitative macrophage infiltration was not as long-lasting as one could expect from the sustained reduction in tumor growth, other effects or immune cells might also be involved. Hayes and colleagues demonstrated that blocking the *cis*-interaction between CD47 and SIRPα both expressed on macrophages plus blockage of CD47 on target cells leads to a kind of hyper-phagocytosis,^36^ which could explain the prolonged antitumoral effect of macrophages due to higher activity. Neutrophils were found to destruct antibody-opsonized cancer cells not by ADCC but rather by trogocytosis, and blocking the CD47-SIRPα interaction further potentiates this activity.^37^ Although CB17-SCID mice lack functional T and B cells, NK cells expressing SIRPα and CD47 are present. Blocking CD47 on NK cells has been demonstrated to increase their antitumoral activity in a syngeneic melanoma model^38^, while blocking CD47 on leukemia cells or major histocompatibility complex (MHC)-I-deficient cells increased the killing capacity of NK cells.^39^ As we could not demonstrate NK staining *in vivo*, we utilized an *in vitro* assay with a genetically modified NK cell line (NK92) overexpressing a polymorphic variant of FcγRIIIa/CD16a, which exhibits the most potent ADCC effect.^40^ For optimal sensitivity, target cells were transfected with the more stable luciferase NanoLuc (nLuc), which gives a bright bioluminescence signal^41^. Lysis activity peaked at E:T ratio 5:1 and 10:1 with ∼50% lysis (Figure S8). This is in line with observations by Shenouda et al., where the parental cell line of 231/LM2-4, namely MDA-MB-231, was efficiently lysed at E:T ratios of 5:1 and above by activated NK cells isolated from healthy donors as well as from breast cancer patients.^42^ Only psCV1-hIgG1-transfected cells were lysed to a significant extent by NK cells (Figure 8). Apparently, a (partial) block of CD47 after transfection with psCV1 (see Figure 2B) is not sufficient to induce NK-cell mediated lysis, and it needs the Fc part bound to the cell surface to trigger FcγRIII binding and subsequent NK cell activation. In our experimental setup, CD47 blockage was occurring on the target cells, but due to the secretion of sCV1-hIgG1, some CD47 blockage on NK cells is possible. Hence, an additional beneficial effect of sCV1-hIgG1 secretion could occur, further increasing antitumoral activity of NK cells.^38^

### Conclusions

In conclusion, it can be stated that within a cancer gene therapy approach the tumor-restricted expression of a secreted, highly effective CD47-blocking protein in conjunction with an Fc part for maximized ADCC and CDC can result in a potent antitumoral response concomitant with activation of both macrophages and NK cells. Shortcomings due to low transfection rates could be easily overcome by the potent bystander effect. Recently, Huang and colleagues published a related gene therapy approach but utilizing an oncolytic adenoviral vector.^43^ Here, the SIRPα-Fc fusion protein was not combined with an IL-2 secretion signal; hence the system relies on the necessity of uncontrolled cell burst and fusion protein release. Another advantage of our approach is the combination of maximal CD47 blockade and immune activation: Although the CV1 variant of SIRPα binds human CD47 with an affinity of 11 pM (i.e., 50,00-fold higher affinity compared to wild-type human SIRPα), it could not be used as a systemically applied IgG1-Fc fusion protein because of exuberated toxicity.^13^ In contrast, the therapeutic fusion protein TTI-621 shows a lower affinity for CD47 (and preferentially binds clustered CD47, hence sparing red blood cells) but comes with a highly active human IgG1-Fc part.^9^ Therapeutic antibodies are currently evaluated in clinical trials (Hu5F9-G4 and SRF231) and have a high CD47 affinity (8 nM for monomeric and 12 pM for dimeric CD47 in the case of Hu5F9-G4^10^) but are of a human IgG4 isotype, which exhibits a 10-fold reduced affinity for human FcγRI and weak ADCC and CDC activity. Currently, Hu5F9-G4 is now also successfully combined with rituximab (anti CD20, IgG1 type) in lymphoma treatment.^44^ Our approach is currently designed for localized, intratumoral application. Its use within a systemically applied, targeted gene delivery system would be the next logical step, where specificity of delivery (e.g., using cell-targeting ligands) and/or specificity of expression (using tumor-specific promoters or miRNA-based de-targeting approaches) is further pursued.^16^^,^^18^^,^^45^ For preclinical studies, syngeneic tumor models need to be evaluated (e.g., CT26 colon cancer in BALB/c mice^14^^,^^46^). This is important as the adaptive immune system, especially CD8+ cytotoxic T cells, is also involved in tumor eradication after CD47 blockage.^47^ Taken together, this gene-based approach can be seen as a platform that should enable blocking of other checkpoint inhibitors with high-affinity and -efficacy fusion proteins, avoiding side effects on non-tumorous tissue.

## Materials and methods

### Cells

MDA-MB-231 (HTB-26, ATCC, Manassas, VA, USA), 231/LM2-4^22^ (kindly provided by R.S. Kerbel, Sunnybrook and Women’s College Health Sciences Centre S-217, Toronto, ON, Canada), and CT26 (CRL-2638, ATCC) were maintained in DMEM, high glucose (Sigma-Aldrich, Vienna, Austria) and A549 (CCL-185, ATCC) in RPMI-1640 (Sigma-Aldrich), all with addition of 1% l-glutamine, 1% penicillin-streptomycin, and 10% fetal bovine serum (FBS; Biowest, Riverside, MO, USA; also termed complete medium), at 37°C, 5% CO_2_. 231/LM2-4-EGFPLuc cells were generated by transduction of 231/LM2-4 cells with a lentiviral vector encoding a PGK-EGFPLuc cassette^16^ and sorted for EGFP expression on a FACS Aria III (BD Biosciences, San Jose, CA, USA) using 488-nm excitation. NK92 cells (PTA8836, ATCC) were maintained in Minimum Essential Medium Eagle (MEM) Alpha Modification (Sigma-Aldrich) supplemented with 100–200 U/mL IL-2, 2 mM l-glutamine, 0.1 mM 2-mercaptoethanol, 0.02 mM folic acid, 0.2 mM inositol, 12.5% horse serum (Cat No H1138, Sigma Aldrich), and 12.5% FBS (Biowest). Treatments under serum-reduced conditions were performed in complete medium containing 1% horse serum and 1% FBS.

### Plasmids and cloning

To generate plasmid psCV1-hIgG1, the sequence IL2-CV1-hIgG1 (1,331 bp) was designed by placing the CV1 sequence^13^ into the multiple cloning site (MCS) of plasmid pFUSE-hIgG1e4-Fc2 (Invivogen, Toulouse, France) situated between the IL-2 secretion signal sequence (s) and the hIgG1e4-Fc sequence (hIgG1). The full sequence IL2-CV1-hIgG1 containing the IL-2 secretion signal sequence, the CV1 sequence, and the hIgG1e4-Fc sequence (hIgG1) was further codon optimized for human expression and to avoid certain *cis*-acting sequence motifs (e.g., internal TATA boxes, RNA instability motifs, and cryptic splice donor and acceptor sites), synthesized, sequenced and cloned into plasmid pMK-RQ by GeneArt (Thermo Fisher, Regensburg, Germany). Furthermore, the IL2-CV1-hIgG1 sequence was cloned into pCpG-hCMV/SCEP-mCherry^18^ using XbaI and NheI replacing the mCherry cDNA. Plasmid psCV1 was generated by excising the IL2-CV1 sequence from psCV1-hIgG1 with BgIII and replacing the mCherry cDNA in pCpG-hCMV/SCEP-mCherry. Plasmid pshIgG1 was generated by excising the CV1 sequence from psCV1-hIgG1 with Eco32I and MbiI. pCpG-hCMV/SCEP-mCherry (for short: pmCherry) was used without further modification.^18^ All plasmids were propagated in *E. coli* DB3.1λpir or GT115 with 25 μg/mL Zeocin, purified with a NucleoBond PC 10 000 Gigaprep Kit (Thermo Fisher Scientific, Austria), and characterized by diagnostic restriction digest. Plasmid pNluc was cloned by inserting the nLuc cDNA from plasmid pNL1.1 (#N1001, Promega, Walldorf, Germany) into pMule_ENTR_CMV-eGFP-L1-R5,^48^ replacing the eGFP sequence in principle as described previously,^49^ and propagated in *E. coli* DH5α with kanamycin.

### Polyplex generation

Polyplexes were generated with 10-kDa LPEI at an N/P ratio (molar ratio nitrogen in LPEI versus phosphate in pDNA) of 9 in principle as described previously.^50^ Equal volumes of a pDNA-LPEI-containing solution were mixed by flash pipetting and incubated for 5 min at room temperature. For *in vitro* experiments, nanoparticles were generated at a final pDNA concentration of 20 μg/mL (of the desired plasmid) in HBS buffer (HEPES-buffered saline, 20 mM HEPES pH 7.4, 145 mM NaCl) and for *in vivo* experiments at 200 μg/mL in HBG buffer (HEPES-buffered glucose, 20 mM HEPES pH 7.4, 5% glucose w/v). Particle size and ζ-potential were measured on a Malvern NS500 system (Malvern, UK) as described previously.^50^

### *In vitro* transfection and bystander effect

3.95 × 10^6^ cells were seeded into T-75 flasks (Sarstedt, Nümbrecht, Germany) 24 h prior to treatment. After removal of medium, polyplexes (40 μg pDNA/flask) were added in 15 mL of basal medium (DMEM, high glucose, serum free) for 4 h, and thereafter 15 mL of complete medium (DMEM, high glucose, 1% l-glutamine, 1% penicillin-streptomycin, 10% FBS) was added. Different polyplex treatment groups were performed depending on the desired plasmid needed for transfection, namely psCV1-hIgG1 (therapeutic plasmid), pshIgG1 (control plasmid; for secreted hIgG1-Fc), psCV1 (control plasmid; for secreted SIRPα), and pmCherry (transfection control plasmid). Transfection supernatant was stored frozen at −80°C until further use. Bystander effect was determined by transferring 30 mL of freshly thawed supernatant (or diluted with complete medium to the indicated ratio) from 48-h-transfected cells to similarly seeded but untransfected cells. These untransfected cells were incubated with supernatant for 1 h at 37°C, and thereafter cells were further processed for analysis. To determine transgene expression over a duration of 12 days, 1.32 × 10^6^ cells were seeded in T-25 flasks, transfected with 13.2 μg plasmid/flask for 48 h (p0), and thereafter further passaged at a ratio of 1:8 after 5 (p1), 9 (p2), and 12 (p3) days into new T-25 flasks with fresh complete medium. Remaining cells were analyzed accordingly for transgene expression.

### Flow cytometry

Cells were detached with TrypLE (A549) or Versene (MDA-MB-231, 231/LM2-4) for 4 min at 37°C, neutralized with complete medium, and washed with phosphate-buffered saline (PBS). Cell count and viability were measured by a flow cytometer (MACSQuant Analyzer 10, Miltenyi Biotec, Bergisch Gladbach, Germany) by adding 1 μg/mL propidium iodide (PI). 150,000 live cells were transferred into 96 V-bottom plates (#M9561, Greiner CELLSTAR, Greiner Bio-One, Kremsmünster, Austria). All antibodies were diluted in 0.5% (w/v) bovine serum albumin (BSA) in PBS (pH 7.4) and indicated amounts in 20 μL dilution added per 150,000 cells. For huCD47 staining, 0.03 μg of mouse-anti-huCD47 (ab3283 clone B6H12.2, Abcam, Cambridge, UK) or purified mouse IgG1, κ isotype control (Cat 557273, BD Biosciences) was added for 30 min at 4°C, followed by three washing steps with PBS and 1-h incubation with 1:2,000 diluted goat anti-mouse IgG H&L (Alexa Fluor 647 [AF647] conjugated, preadsorbed; ab150119, Abcam). For Fc detection, cells were incubated with 1:2,000 diluted protein A-AF488 conjugate (P11047, Thermo Fisher Scientific, Vienna, Austria), 1:500 diluted goat anti-human IgG Fc (DyLight 650 conjugated, preadsorbed; ab98622, Abcam), or 1:1,000 goat IgG isotype control AF647 conjugate (Bs_0294P-A647, Bioss Antibodies, Woburn, MA, USA) for 1 h at 4°C, washed three times with PBS, and diluted in 0.5% BSA-2 mM EDTA-PBS for the measurement performed on a MACSQuant Analyzer 10 Flow Cytometer (Miltenyi Biotec). Live/dead cell staining was performed with 4¢,6-diamidino-2-phenylindol (DAPI) (1 μg/mL) and cells gated for live cells. Data analyses were done with FlowJo software (version 1.10).

### Dot blot analysis

Transfection supernatant (2 μL/dot) was dropped onto a nitrocellulose membrane (sc-201706, 0.45 μm; Santa Cruz Biotechnology, Heidelberg, Germany) and air-dried; a standard curve was generated similarly with human IgG1 isotype (ALX-804-133-C100; Enzo Biochem, Farmingdale, NY, USA). BSA (Sigma-Aldrich; 2 mg/mL) and complete medium were dotted as negative controls. The membrane was washed twice with TBS-T (Tris-buffered saline with 1% [w/v] Tween 20) and incubated for 1 h with anti-human IgG Fc-horseradish peroxidase (HRP) (1:10,000 dilution in TBS-T; ab98624, Abcam) at room temperature. After three washing steps with TBS-T, the blot was incubated for 1 min with enhanced chemiluminescence (ECL) substrate (50 μL luminol, 22 μL p-coumaric acid, and 3 μL H_2_O_2_ [33%] in 10 mL 100 mM Tris pH 8.5) and chemiluminescence was recorded on a Molecular Imager Gel DocTM XR System (Bio-Rad, Vienna, Austria). The integrated signal density was evaluated via ImageJ (ImageJ 1.53a) and data evaluation performed with Microsoft Excel (version 2013) and GraphPad Prism (version 6.0).

### Confocal laser scanning microscopy

25,000 cells/well were seeded into chambered slides (Nunc Lab-Tek II 8-well slides; Thermo Fisher Scientific) 72 h prior to treatment and then washed with PBS, and 400 μL of 48-h transfection supernatant (1:2 dilution in complete medium) was added for 1 h at 37°C. After PBS washing (3 times) cells were fixed with formaldehyde (4% w/v, in HBS) for 40 min, PBS washed (3 times), and stained for 1 h with goat anti-human IgG Fc (DyLight 650, preadsorbed; ab98622) diluted 1:200 in PBS + 0.5% BSA. After PBS washing and DAPI staining (1 μg/mL), cells were imaged on a Leica TCS SPE microscope (Leica, Wetzlar, Germany) with a 40× oil immersion objective with laser excitation (405 nm for DAPI, 635 nm for DyLight 650) and appropriate emission filters. Z-scans with a vertical resolution of 0.1 μm were conducted and central sections shown. Data acquisition and analysis was done with LAS X software (version 3.1.2.16221).

### NK cell co-culture cell lysis assay

3.95 × 10^6^ 231/LM2-4 cells were seeded in T-75 flasks and 24 h thereafter co-transfected with polyplexes containing 40 μg of pNluc and 40 μg of the indicated plasmid (psCV1-hIgG1, psCV1, or pshIgG1 or pmCherry) for 4 h in basal medium; thereafter, complete medium was added and after a total of 48 h cells were harvested and transferred to 48-well plates (Greiner Bio-One; 100,000 cells/well). After a further 24 h of growth, medium was removed and NK92 cells were suspended in 200 μL/well serum-reduced medium at E:T ratios of 10:1, 5:1, 2:1, and 1:1 or medium only (w/o co-culture) added for 4 h at 37°C. The supernatant was harvested and centrifuged for 5 min at 300 × *g*, and nLuc luciferase released into supernatant was measured in white 96-well plates (Cat No 655098; Greiner Bio-One) (40 μL/well) with the Nano-Glo Luciferase Assay System (#N1130, Promega) analyzed on a GM2000 GloMax Navigator Microplate Luminometer (Promega, software version 3.0). Cells treated with medium containing 0.1% (v/v) Triton X-100 served as positive control. Relative NK cell-mediated lysis was calculated with the formula NK cell cytotoxicity (%) = 100 × (nLuc (experimental) − nLuc(w/o coculture))/(nLuc (Tx100) − nLuc (w/o coculture)).

### *In vivo* studies

Female CB17-SCID mice (CB-17/Icr-Prkdcscid/scid/Rj; Janvier Labs, Le Genest-Saint-Isle, France) were kept in individually ventilated type 2l cages with food and water provided *ad libitum*. All animal procedures were approved by local ethics committee and are in accordance with the Austrian law for the protection of animals and EU Directive 2010/63/EU. 1 × 10^6^/30 μL of 48-h-transfected 231/LM2-4-EGFPLuc cells resuspended in sterile PBS was percutaneously implanted into the 4^th^ pair of inguinal nipples (two implantation sites), and tumor growth was evaluated by BLI as described below. In a separate experiment, intratumoral injection into the middle of the untransfected tumor core (two puncture sites per tumor) was performed on day 13 after implantation (50 μL polyplex solution, 200 μg/mL pDNA) with a 26 g needle and a 2-mL syringe (Braun, Melsungen, Germany). BLI signal was detected after injection of d-luciferin (potassium salt; Intrace Medical, Lausanne, Switzerland; 30 mg/mL in DPBS; dosage: 120 mg/kg) with an IVIS Spectrum-CT imaging system (PerkinElmer) in principle as described previously^50^ and quantified by placing appropriate regions of interest (ROIs) onto tumor sites.

### Immunohistochemistry

Tissues were fixed for 22 h in 4% formaldehyde in HBS and further processed as described previously.^50^ For CD47 staining, sections were pre-warmed for 2 h at 55°C, deparaffinized, and dehydrated, and heat-induced epitope retrieval (HIER) was performed for 30 min in Tris-EDTA buffer (10 mM Tris, 1 mM EDTA, pH 9). Slides were treated with normal goat serum (30 min) and avidin/biotin blocking reagent (BUF016, Bio-Rad) for 15 min each, followed by incubation with rabbit-anti-huCD47 antibody [EPR21794] (ab218810, Abcam; 1:2,000) or Rabbit IgG, monoclonal [EPR25A]-Isotype Control (ab172730, Abcam) in PBS + 2% BSA overnight at 4°C. Endogenous peroxidase was blocked with 0.3% H_2_O_2_-PBS for 15 min and signals detected via Vectastain ABC HRP Kit (Peroxidase, Rabbit IgG, PK-4001; Vector Laboratories, Burlingame, CA, USA). For F4/80 staining, slides were incubated in 0.3% H_2_O_2_-PBS for 10 min, heated in DAKO antigen retrieval solution (pH 6.0, #1699, Agilent, Santa Clara, CA, USA) for 1 h, and blocked for 5 min with 10% goat serum-0.1% Tween 20 in PBS. Incubation with rat anti-mouse F4/80 (#123102, BioLegend, San Diego, CA, USA) diluted 1:25 in 1% goat serum-PBS overnight at 4°C was followed by labeling with biotinylated goat anti-rat antibody (#BA9401, Vector Laboratories) and staining with the Vectastain Elite ABC Kit (#PK6100, Vector Laboratories) (30 min each) and staining with 3,3¢-diaminobenzidine (DAB). Slides were counterstained with hematoxylin and mounted in Entellan new. Numbers of F4/80-positive cells/field were evaluated by Definiens analyses (Definiens, Munich, Germany). Images were acquired on an Olympus BX53 microscope.

### Statistical analysis

Statistical analysis was performed with GraphPad Prism 6 and an online tool (https://www.socscistatistics.com/). For the comparison of two treatment groups a U test (Mann-Whitney) was performed. Every effort was made to keep testing consistent across related experiments.

## Supplemental information

Document 1. Figures S1–S8

Document 2. Article plus supplemental information
