# Supplementary material for: CD47-targeted cancer immunogene therapy: Secreted SIRPα-Fc fusion protein eradicates tumors by macrophage and NK cell activation
Source: Mol Ther Oncolytics. 2021 Oct 1;23:192–204. doi: 10.1016/j.omto.2021.09.005 (PMC8526499; doi:10.1016/j.omto.2021.09.005)
Supplement: Document 1. Figures S1–S8 [file mmc1.pdf]

OMTO, Volume *23*

Supplemental information

CD47-targeted cancer immunogene therapy: Secreted SIRPα-Fc fusion protein eradicates tumors by macrophage and NK cell activation

Magdalena Billerhart, Monika Schönhofer, Hemma Schueffl, Wolfram Polzer, Julia Pichler, Simon Decker, Alexander Taschauer, Julia Maier, Martina Anton, Sebastian Eckmann, Manuel Blaschek, Petra Heffeter, Haider Sami, and Manfred Ogris

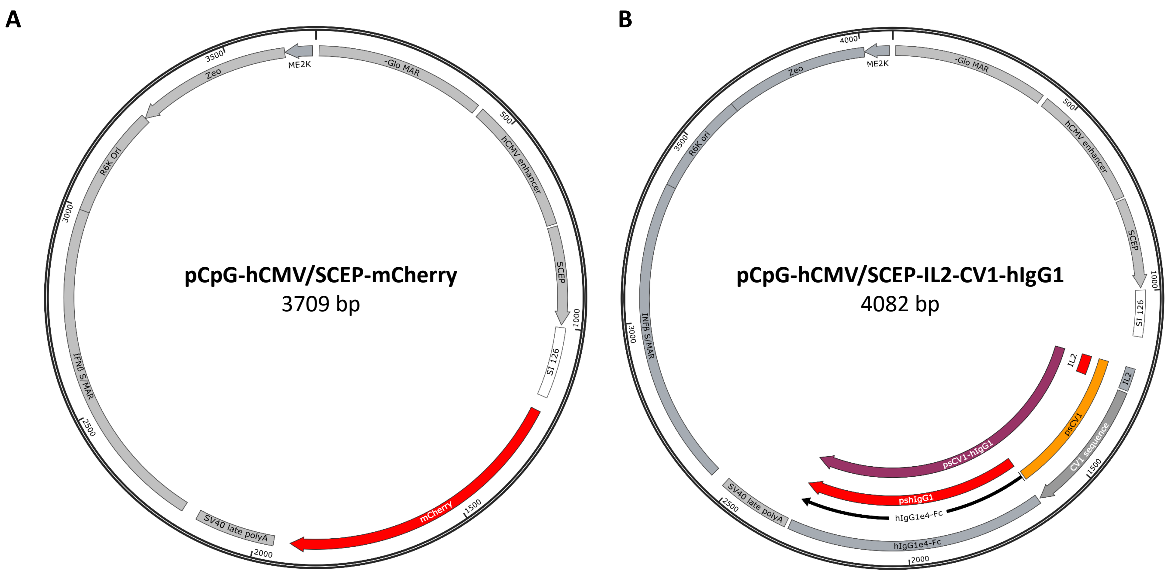

Fig. S1. Maps of plasmids used in the study.

(A) pmCherry (full name: pCpG-hCMV/SCEP-mCherry), this was used as the transfection control plasmid as it shares the same backbone as for all cloned plasmids but with a mCherry sequence for reporting purposes (indicated in red). For all other plasmids, this mCherry sequence is just replaced by the respective fusion protein sequence. (B) Plasmid map of the therapeutic plasmid (psCV-hIgG1; full name: pCpG-hCMV/SCEP-IL2-CV1-hIgG1 showing its components in violet). Single-component control plasmids just differ in the sequence for the respective secreted fusion protein i.e. encoded by psCV1 (orange) or pshIgG1 (red).

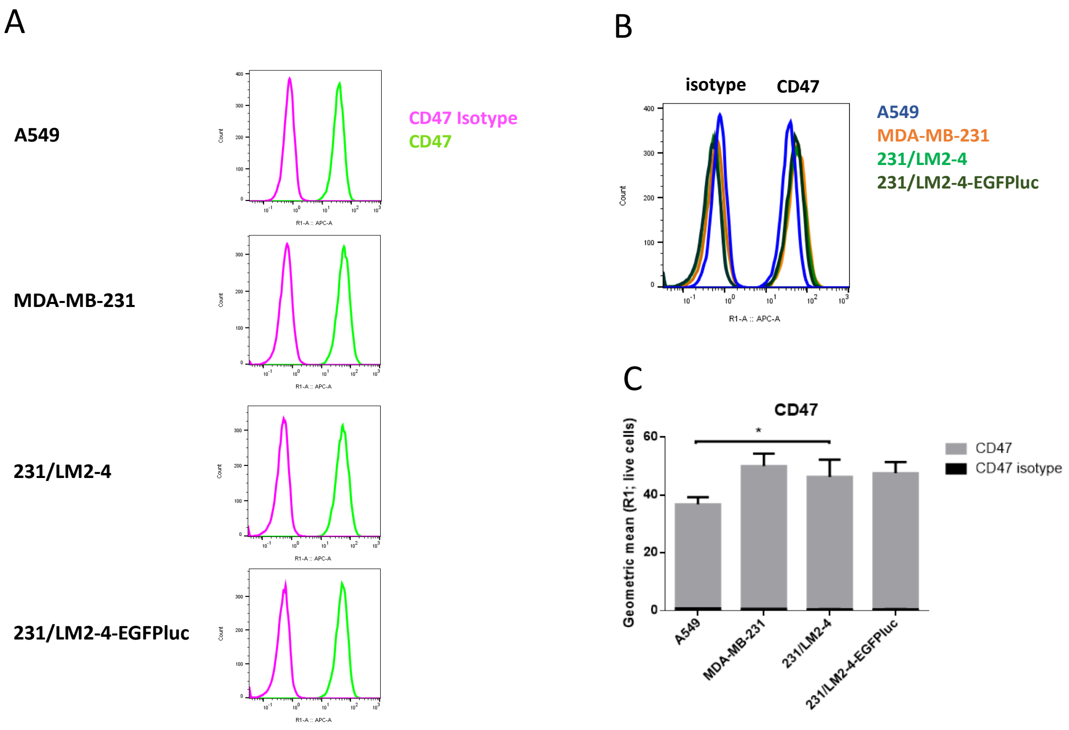

Fig. S2. CD47 expression in different human cancer cell lines used in this study.

(A) Individual histogram plots for indicated cell lines, CD47 staining (clone [B6H12.2], Abcam) vs isotype control (purified mouse IgG1, κ isotype control, BD Biosciences), secondary antibody goat anti-mouse IgG H&L - Alexa Fluor® 647; (B) histogram overlay CD47 staining vs isotype control; (C) geometric mean of individual measurements of CD47 signal from indicated cell lines, n= 6; *P < 0.05 A549 vs 231/LM2-4; u-test (Mann-Whitney).

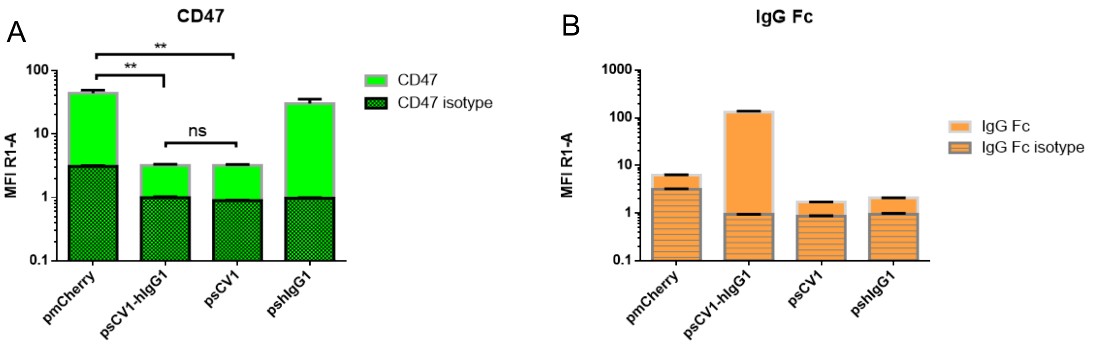

Fig. S3. CD47-blockage and sCV1-hIgG1-binding on in vitro transfected A549 cells

A549 cells were transfected in T-75 flasks with the indicated plasmid for 4 h in basal medium, thereafter same amount of complete medium added, harvested after 48 h and analyzed by flow cytometry. CD47-blockage was evaluated by staining with anti-CD47 antibody [B6H12.2] competing with sCV1-hIgG1 for CD47-binding and isotype IgG staining as control (secondary antibody goat anti-mouse IgG H&L Alexa Fluor® 647). sCV1-hIgG1-binding to the cell surface was detected by goat anti-human IgG Fc - DyLight® 650 staining or goat IgG isotype AF647 conjugate. (A) CD47 stain; (B) IgG-Fc stain; average signal intensity (geometric mean of individual measurements); n=6, ** p≤ 0.01, ns p>0.05; u-test (Mann-Whitney).

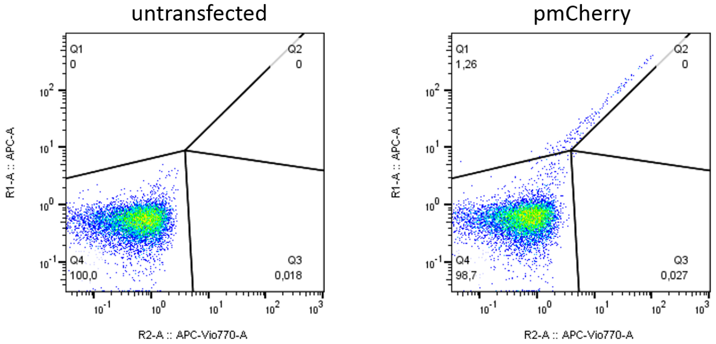

Fig. S4. Transfection efficiency in vitro

231/LM2-4-EGFPluc cells were transfected in T-75 flasks with pmCherry for 4 h in basal medium, thereafter the same volume of complete medium added, harvested after 48 h and analyzed by flow cytometry (ex. 640 nm) using diagonal gating (x-axis: Emission 750 nm longpass, y-axis: Emission 655-730 nm); 10,000 live cells (after DAPI live/dead staining) are shown, left panel: untransfected cells, right panel: pmCherry transfected cells; Q1: mCherry positive cells (%).

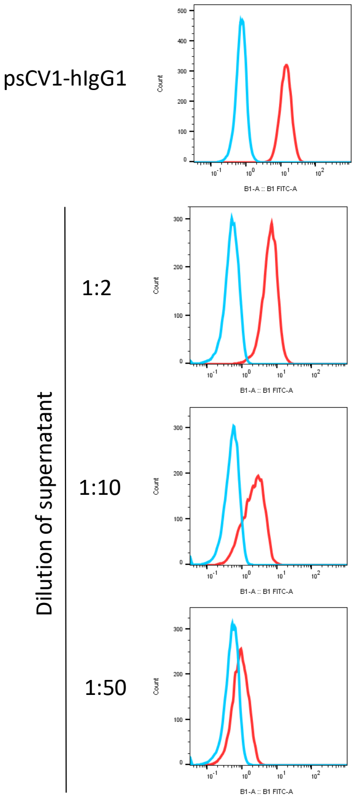

Fig. S5. sCV1-hIgG1 is expressed by murine tumor cells and binds to the cell surface

CT26 cells were transfected for 48 h with psCV1-hIgG1, harvested, stained with protein A-AF488 and analyzed by flow cytometry (top panel). Cells treated with 48 h-transfection supernatant for 1 h (diluted with complete medium at the indicated ratio) and thereafter stained with protein A-AF488; red line: sCV1-hIgG1-transfected cells or cells treated with transfection supernatant; blue line: untreated control cells or mock-transfected cells.

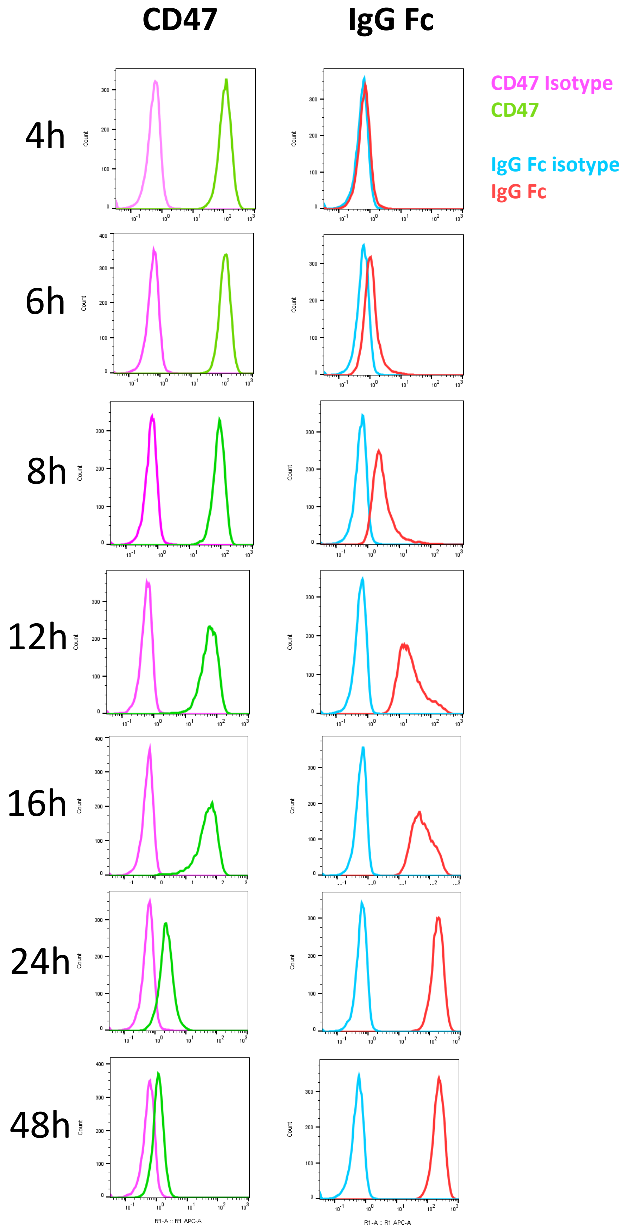

Fig. S6. Time course of transgene expression in vitro, histogram analysis

231/LM2-4 cells were transfected with psCV1-hIgG1 for 4 h, 6 h, 8 h, 12 h, 16 h, 24 h and 48 h as described in materials and methods, thereafter, harvested and analyzed by flow cytometry. For CD47 detection (left column), cells were stained with anti-CD47 antibody [B6H12.2] or IgG isotype (secondary antibody goat anti-mouse IgG H&L Alexa Fluor® 647); for Fc detection (right column), cells were stained with goat anti-human IgG Fc - DyLight® 650 or goat IgG isotype AF647 conjugate.

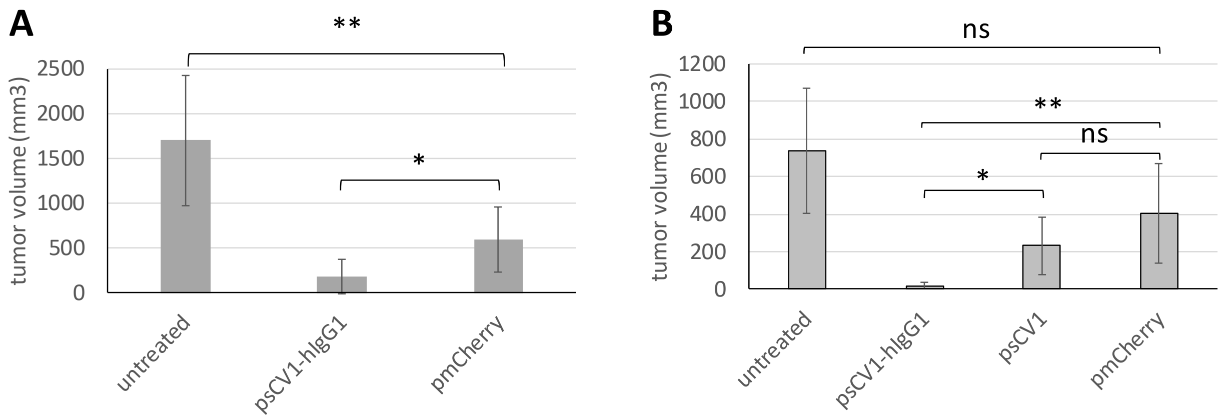

Fig. S7. Tumor volumes of ex vivo transfected 231/LM2-4-EGFPLuc tumors

231/LM2-4-EGFPLuc cells were transfected with the indicated plasmid as described in Fig 5 or left untreated and thereafter orthotopically implanted into the 4th pair of inguinal nipples (2 sites/animal) into female CB17-SCID mice and tumor growth followed by BLI measurements. Experiment was terminated on (A) day 34 (Exp I) or (B) day 25 (Exp II), tumors were explanted, fixed in formalin and the volume calculated after measuring length and width of the tumor by the formula volume = (width^2 × length)/2; n=6; ** p≤ 0.01, * p≤0.05; ns not significant; U-test (Mann-Whitney), two-tailed.

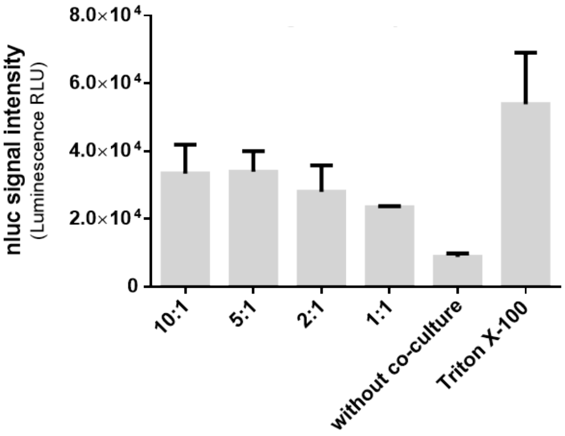

Fig. S8. NK cell mediated cell lysis of pNluc transfected 231/LM2-4 cells

231/LM2-4 cells were transfected with plasmid pNluc (40 µg per T-75 flask) for 48 h, transferred to 48-well plates and 24 h thereafter co-cultured with NK-92 cells at the indicated E:T ratio for 4 h. Cells treated with medium containing 0.1% Triton X-100 served as positive control, cells without co-culture as negative control. Nluc activity was quantified in the supernatant; n = 6 + stddev.
